# Organochlorine pesticides residue affinity in fish muscle and their public health risks in North West Ethiopia

**DOI:** 10.1002/fsn3.3025

**Published:** 2022-08-09

**Authors:** Birhan Agmas Mitiku, Marshet Adugna Mitiku

**Affiliations:** ^1^ Department of Veterinary Science, College of Agriculture and Environmental Science Bahir Dar University Bahir Dar Ethiopia; ^2^ Ethiopian Institute of Agricultural Research National Fishery and Aquatic Life Research Center Sebeta Ethiopia; ^3^ Addis Ababa University Aklilu Lemma Institute of Pathobiology Addis Ababa Ethiopia

**Keywords:** fish, muscle, pesticide, public health risk, residues

## Abstract

Pesticides are the parent compounds, their metabolites, and associated impurities of agricultural and health chemical inputs. If they are found at concentration levels higher than the standard limits, they have potential negative impacts on the ecosystem in general and on fish and humans in particular. This study investigates organochlorine pesticides (OCPs) residue occurrences in fish muscle and assesses their public health potential risks, in North West Ethiopia. The concentration of OCPs residue under gas chromatography with electron capture detector (GC‐ECD) was detected in 37.84% of fish muscle samples. The mean amounts detected were Endosalfan I, 341.50 ± 32.19 μg/kg; Endosalfan II, 36.01 ± 2.3 μg/kg; Endosalfan sulfate, 5.43 ± 4.06 μg/kg; 4, 4, DDE (4,4‐dichlorodiphenyldichloroethylene), 64.01 ± 9.08 μg /kg; 4,4, DDD (4,4‐dichlorodiphenyldichloroethane), 5.65 ± 3.12 μg/kg; and 4, 4, DDT (4,4‐dichlorodiphenyltrichloroethane), 1.58 ± 0.30 μg/kg. The mean concentration of Endosalfan I tested in fish muscle samples was higher than that of the permissible limit of different international standards. However, due to the low per capita consumption rate of fish origin food in Ethiopia, the health risk index (HRI) ranges from 0.002 to 0.1275, which shows there is no public health risk. This study highlights the possibility of chemical residue occurrence in fish food products, and hence pesticide use regulations and monitoring concentration levels should be implemented regularly to avoid human and environmental health risks.

## Graphical abstract

This study investigates organochlorine pesticides (OCPs) residue occurrences in fish muscle and assesses their public health potential risks, in North West Ethiopia. It also highlights the possibilities of OCPs residue occurrences in fish food products. Hence, pesticide use regulations and monitoring concentration level should be implemented to avoid human and environmental health risks.

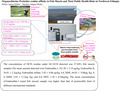

## 1 INTRODUCTION

Pesticides are chemicals that can contaminate the aquatic and terrestrial environments. It is known that pesticides applied in an area reach into the aquatic environment through drift, leaching, and drainage (Srivastava et al., 2010). Pesticides, mainly organochlorine pesticides (OCPs), are poorly hydrolyzed and slowly biodegrade in the environment. This chemical nature of OCPs contributes to their wide distribution in the environment, and thus they are considered to be significant chemical food contaminants. These OCPs ultimately biomagnify in various trophic levels and eventually cause high burden in fish and consequently threaten the health of humans (Donaldson et al., 2010; Khodadadi et al., 2012; Srivastava et al., 2010).

The risks associated with consumption of animal‐origin food should be taken into account in the agricultural sector by reasonable and achievable good practices in the agricultural industry. The maximum levels of OCPs residues in foodstuffs of animal origin should be set at the strictest possible level due to the lipophilic nature of these chemicals (Fair et al., 2018; USEPA, 2009). According to the WHO/FAO (2009), humans, animals, and fishes are considered at risk when they consume OCPs residues at levels higher than the maximum residue limits and acceptable daily intake.

Exposure to OCPs compounds is especially dangerous during prenatal development and infancy, as it causes irreversible changes in the central nervous system. OCPs have been shown in studies to have a high potential to cross placental barriers and cause serious neonatal damage even at low concentrations (Jayara et al., 2016; Thompson et al., 2017). Long‐term health consequences include an increased risk of reproductive disorders, kidney and liver dysfunction, nervous system and birth defects, endocrine disruption, immune system dysfunction, and carcinogenesis (Okoffo et al., 2016; Yazgan & Tanik, 2010). Besides, it causes cardiovascular diseases, disrupts heme biosynthesis and vitamin D metabolism, and disrupts mineral metabolism (Jayara et al., 2016).

The problem of OCPs stems not only from their toxic properties but also from low levels of exposure to these chemicals. Their effects can be observed only at the physiological or biochemical level. The acute effect of OCPs is the intoxication of fish, humans, and other animals by direct contact or through the food chain (Jayara et al., 2016; Thompson et al., 2017). Numerous studies on both humans and animals provide strong evidence of the toxic potential of OCPs (Ravindran et al., 2016). According to the study conducted by Yazgan and Tanik (2010), the acute toxicity effect of OCPs causes environmental risks that may cause ecological damage, including to the flora and fauna of the entire ecosystem.

According to various studies, the level of OCPs in developing countries is increasing as it is still used for agriculture and public health purposes, while it is declining in developed countries (Sadasivaiah et al., 2007; Thompson et al., 2017; WHO, 2010). Ethiopia has a relatively well‐developed pesticide legislation on registration and control of pesticides intended to address their environmental and health effects (Negatu et al., 2016). In addition, Ethiopia is a signatory to the Stockholm Convention and agreed to support research on persistent organic pollutants. But there are gaps between policy and practical enforcement of prohibiting the import and application of banned OCPs including DDT and Endosulfan (Negatu et al., 2016). However, limited data are available about the occurrence and level of pesticides in the aquatic environment, specifically in fish tissue. In Ethiopia, studies in rift valley water bodies have revealed the contamination of the environment (sediment) and fish species (*Oreochromis niloticus*, *Clarias gariepinus*, *Tilapia zilli*, and *Carrassius*spp.) by pesticides (Yohannes et al., 2014). The study conducted by Deribe et al. (2014) indicated that *Barbus intermedius*, *O. niloticus*, and *C. gariepinus* were found to be contaminated by different pesticides. Ethiopian environmental health issues, especially OCPs, request further efforts of scholars for incessant investigation to draw a clear map of the situation (Thompson et al., 2017).

The first version of this study indicates that there is still an increasing trend of OCPs applications for improving agricultural production in the catchments of Lake Tana (Agmas & Adugna, 2020). Accordingly, high concentrations of OCPs can be found in the environment and fish muscle of our study area. In Ethiopia, the studies conducted to assess the contamination of OCPs in the environment are scarce; none has been conducted in Lake Tana. Dried fish products are being exported to neighboring countries, especially to the Republic of Sudan and Eritrea recently. Hence, the research result will help to amend fish product export policies and associated environmental safety considerations regionally and locally. Therefore, the aim of this study was to assess OCPs accumulation in the muscle of fish and estimate its potential human health risks in fish of Lake Tana, North West Ethiopia.

## 2 METHODOLOGY

### 2.1 Study area

The study was conducted in Lake Tana, the headwaters of the Blue Nile River. It is located in the province of North West Ethiopia. It is located approximately 580 km north west of the city of Addis Ababa. The lake is one of the largest (3600 km^2^) and best fishing sites in Ethiopia. Geographically, Lake Tana is found at latitude 12°1'35.75"N and longitude 37°18'12.54"E. Mean annual temperatures range from 13 to 22°C. The annual average rainfall of Lake Tana is 1248 mm per year (Stave et al., 2017).

**FIGURE 1:**
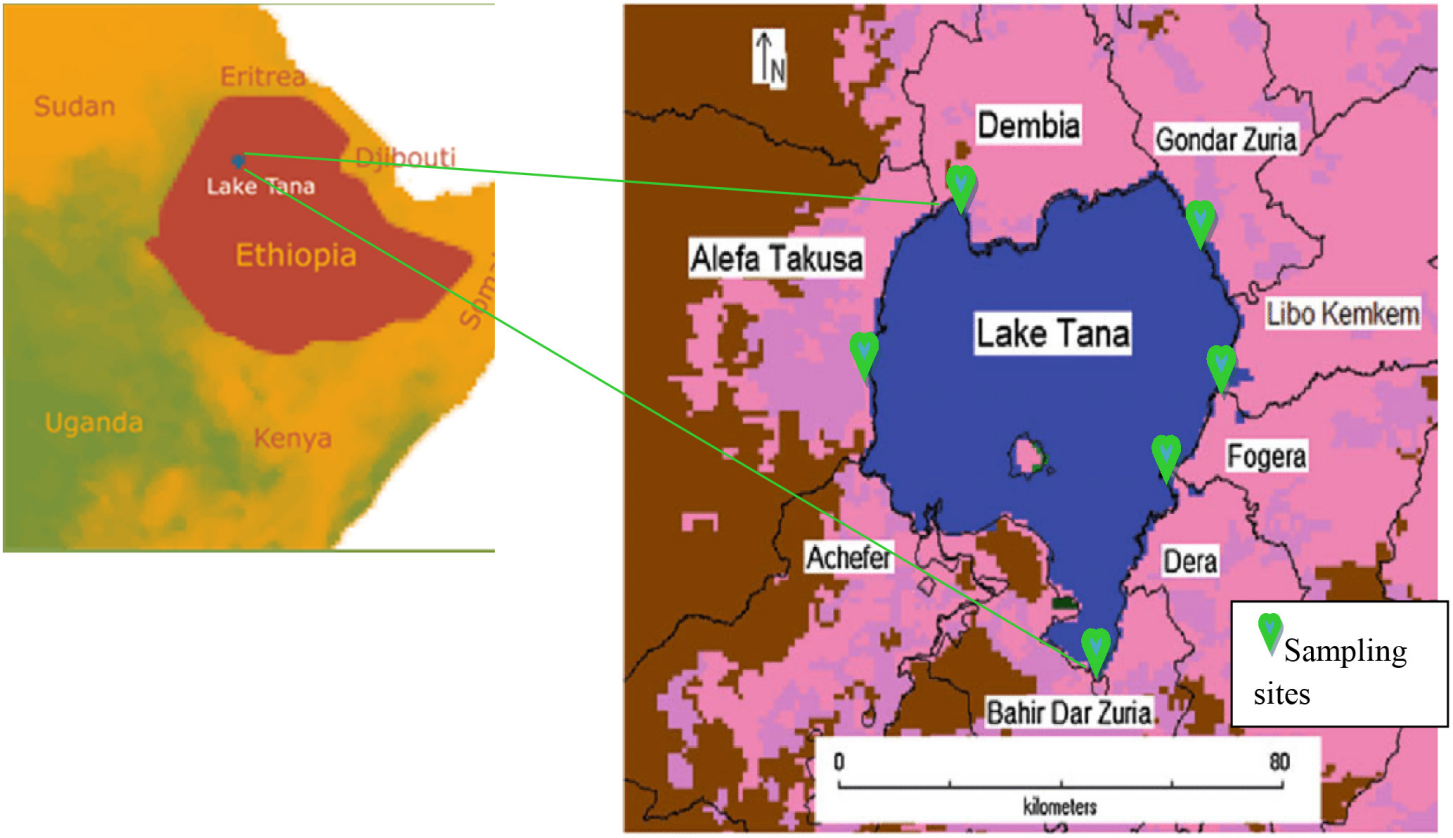
Map of the study area (Source: Modified map from spatial data in DIVA‐GIS 2015).

The lake has three main commercially important fish groups, such as: *Labeobarbus intemedius*, *Clarias gariepinus*, and *Oreochromis niloticus*. They are consumed by larger part of the community and traded widely in the region, even to neighboring Sudan in dry form. There are 55 fishing enterprises and a total of 21,084 beneficiaries directly dependent on fishing activities (Mengistu et al., 2017; Shewit et al., 2018). The samples were collected from six representative Lake Tana landing sites (Gorgora, Enfranze, Kidsthana, Delgie, Agede‐kergna, and Bahir Dar; Figure 1).

### 2.2 Study design and sampling method

The cross‐sectional study design was carried out from January 2018 to December 2020. Three commercially important fish species of the lake, including *Labeobarbus intermedius*, *Clarias gariepinus*, and *Oreochromis niloticus* muscle tissue, were sampled for this study. A total of 137 fish were sampled, with an average size of 446 g for *O. niloticus*, 675 g for *C. garipinus*, and 378 g for *L. intermedius*. Muscle tissue was taken from the dorsolateral sides of each sampled fish. The samples were wrapped in aluminum foil, packed in clean polyethylene bags, labeled and sealed, and then transported in a thermo‐insulated container with ice packs.

### 2.3 Laboratory procedures

#### 2.3.1 Sample extraction and clean ups

Gas chromatography (GC) was used for the pesticide residue analysis. The gas chromatography with electron capture detector (GC‐ECD) processing and analysis were conducted in the laboratories of JIJE analytical and testing service laboratory, Nifas silk, Lafto Sub‐city, Addis Ababa, Ethiopia. All glassware and containers were washed with detergent, rinsed with purified water and acetone, and kept in an oven at 180°C for 2 h for processing (Anastassiades et al., 2003).

The extractions were carried out according to the quick, easy, cheap, effective, rugged, and safe (QuEChERS) method described by Anastassiades et al. (2003), with some necessary modifications. Lyophilized dry fish muscle sample was chopped on a chopping board with a sharp knife and ground using a mini‐chopper, then 10 g of the chopped sample was transferred to a 50 ml Teflon centrifuge tube. Then, 10 ml of acetonitrile was added to the centrifuge tube and agitated well for proper mixing, followed by the addition of 7.5 g of anhydrous MgSO_4_ and 1 g of NaCl, and the centrifuge tube was vigorously shaken for 1 min. Later, it was centrifuged at 5000 rpm for 5 min. After centrifugation, 2 ml of the supernatant was transferred to an Eppendorf tube containing 100 mg of primary secondary amine (PSA), 150 mg of MgSO_4_, and 100 mg of charcoal for cleanup, followed by vigorous shaking for 2 min. Again, the prepared sample extract was centrifuged at 10,000 rpm for 5 min. Afterward, the sample extract was filtered through a 0.45‐μm filter using a syringe and transferred to auto‐sampler vials for further gas chromatography (GC) analysis.

#### 2.3.2 GC analysis

The OCPs residues were analyzed by a Shimadzu GC‐2010 with an electron capture detector (ECD), an auto‐injector (AOC‐20i; Shimadzu, Kyoto Prefecture, Japan), and GC solution software. The capillary column used in ECD was Rtx‐CL, 30.0 m length/0.25 mm in diameter 0.32‐μm film thickness. The GC was run under the following conditions: injector temperature: 250°C; detector temperature 330°C; oven temperature: 260°C starting from 0 to 180°C for 0.3 min and continued at 5°C/min to 220°C, held for 12 min, and continued at 5°C/min to 260°C; injected sample volume: A 1–2‐μl mode of injection: split; carrier gas: N_2_ with a 77.8 kPa flow rate; runtime: 28 min. Standard peaks were detected by inserting a high concentration of the standard (1 ppm), and the retention time for OCPs was estimated.

#### 2.3.3 GC calibration standards

The GC quantification for individual OCPs was performed using external standard calibration because of the simplicity and sensitivity of the ECD. The lowest concentration calibration standard analyzed during initial calibration was established (the lowest detection limit was 0.001 μg/kg). Commercially prepared stock standard solutions of OCPs (98%–99% purity) that are certified were used (purchased from Thermo Fisher Scientific, USA). Thus, individual standard stock solutions (100 mg/L) were kept in a brown bottle ready for use.

##### Calibration factor

Sample peak areas (or peak heights) are compared to the peak areas (or heights) of the standards. Our external standard calibration was employed by calculating the calibration factors for each analyte at each concentration, the mean calibration factor, and the relative standard deviation (RSD) of the calibration factors, using the formulas below.

Calculate the calibration factor for each analyte at each concentration as:

$$\mathrm{CF}=\frac{\text{Peak Area}\;\left(\text{or Height}\right)\;\text{of the Compound in the Standard}}{\text{Mass of the Compound Injected}\;\left(\text{in nanograms}\right)}$$

Calculate the mean calibration factor for each analyte as:

$$\text{Mean}\;\mathrm{CF}=\frac{\sum_{i=1}^{n}\mathrm{CFi}}{n}$$

Where *n* is the number of standards analyzed.

Calculate the standard deviation (SD) and the RSD of the calibration factors for each analyte as:

$$\mathrm{SD}=\frac{\sqrt{\sum_{i=1}^{n}{\left(\mathrm{CFi}-\mathrm{CF}\right)}^{2}}}{n-1}$$

$$\mathrm{RSD}=\frac{\mathrm{SD}}{\mathrm{CF}}\times 100$$

If the RSD for each analyte was <20%, then the response of the instrument was considered linear fitted model and the mean calibration factor may be used to quantify sample results. Each analyte in each subsequent standard run during the 12‐h period (analytical shift) must fall within its respective retention time window.

The amount of sample for each chromatogram component peak that corresponds to the compounds used for calibration was calculated as follows. The appropriate selection of a baseline from which the peak area or height was necessary to determine for proper quantification:

$$\text{Concentration}\left(\mathrm{\mu g}/\mathrm{kg}\right)=\frac{\left(A_{x}\right)\left(V_{t}\;\right)\left(D\right)}{\left(\mathrm{CF}\right)\left(V_{i}\;\right)\left(W_{s}\right)}$$

Where, *A*_*x*_, Area (or height) of the peak for the analyte in the sample; *V*_*t*_, Total volume of the concentrated extract (μl); *D*, Dilution factor, however we did not make dilution, *D* = 1; CF, Mean calibration factor from the initial calibration (area/ng); *V*_*i*_, Volume of the extract injected (μl). The injection volume for samples and calibration standards was the same; *W*_*s*_ = Weight of the sample extracted (g). The dry weight was used. Since we use kilograms, we multiply our results by 1000.

Using the units given here for these terms resulted in a concentration in units of ng/g, which is equivalent to μg/kg.

Each OCP was measured by comparing the peak areas of the OCPs in the samples and standard, and each OCP was recognized by comparing the elution times of standard OCPs with those in the samples. The concentrations of OCPs were determined from a 6‐point (range of 0.01–10 μg/L) calibration curve of each tested compound. Plots of the integrated peak regions versus concentration were made. The concentrations of each component were run 15 times, and the correlation coefficient (*R* ^2^) for each compound was determined in order to verify the linearity of the calibration plots. For quality control purposes, we analyzed standards after every 20 samples to prevent the number of samples being re‐injected when the standards fail the quality control limits.

### 2.4 Public health risk calculation

Comparison of observed concentrations of pollutants in the environment with established maximum permissible levels (MPLs) is the basic approach used to assess the potential risk posed to ecosystems and human health by toxic effects of pollutants. Irrespective of the eating habits and consumption rate, Food and Agriculture Organization (FAO) and World Health Organization (WHO) recommend the acceptable daily intake (ADI) to assess human exposure to target contaminants. An individual's exposure to OCPs residues from the fish used for the study was achieved by calculating the estimated daily intake (EDI) in μg/kg body weight/day of OCPs by the equation:

$$\mathrm{EDI}=\frac{C\times D}{B}$$

where *C* represents the concentration of OCPs residues in fish (μg/kg) on dry weight basis, *D* the average daily intake of fish estimated at 0.41 g/person/day for adults, and *B* the average body weight considered to be 55 kg for adults. To ascertain the potential public health risk of these estimated exposures, exposure values were compared to two benchmark concentrations for cancer and noncancer health effects. These benchmarks were founded on standard toxicological references. A benchmark concentration represents the daily concentration of a contaminant below which there is a high probability of no adverse health effect (Dougherty et al., 2010; Liu et al., 2010). Health risk assessment of consumers from the intake of OCPs contaminated fish was characterized by using health risk index (HRI). According to WHO/FAO (2009), the estimated HRIs were obtained by dividing the EDI by their corresponding values of acceptable daily intakes (ADI), as shown by the equation;

$$\mathrm{HRI}=\frac{\mathrm{EDI}}{\mathrm{ADI}}$$

The food concerned is acceptable when the HRI is <1 and is risk to the consumer if it is >1, indicating that there is a high probability of adverse health effect (Akoto et al., 2016).

### 2.5 Data analysis

The data were entered into an excel spreadsheet, cleaned by EPI‐Info V.3.5.3, and exported via state transfer to be cleaned, edited, and analyzed by SPSS version 20. Data cleaning was performed to check for accuracy, consistencies, and missed values. A multiple logistic regression analysis model was used to assess the association between pesticide residue occurrence in fish meat and risk factor variables. The adjusted odds ratio (OR) at a 95% confidence interval (CI) value, not including 1, was considered statistically significant.

## 3 RESULTS AND DISCUSION

### 3.1 Occurrence of pesticide residues in fish meat

Overall, gas chromatography (GC) laboratory analysis of the 137 fish muscle samples revealed 37.84% were positive for OCPs. This analysis study indicated that six pesticide residues, including Endosalfan I, Endosalfan II, Endosalfan sulfate, 4, 4, DDE, 4, 4, DDD, and 4, 4, DDT residues, were detected (Table 1). This study's finding was different from that of Gustav Gbeddy et al. (2012), who found OCPs in 100% of the tested samples, which is higher than that of the present study. Another higher frequency of occurrences of OCPs detected by Bedigama and Gabadage (2018) in Sri Lanka was 80%.

**TABLE 1:** Organochlorine pesticides (OCPs) detected in Lake Tana fish muscle (*n* = 137)

No	OCPs	Range (μg/kg)	Overall mean ± SD (μg/kg)
1	Endosalfan I	0.001–4041.73	341.50 ± 32.19
2	Endosalfan II	ND–628.27	36.01 ± 2.3
3	Endosalfan sulfate	ND–146.69	5.43 ± 4.06
4	4, 4, DDE	ND‐227.11	64.01 ± 9.08
5	4, 4, DDD	ND–55.59	5.65 ± 3.12
6	4, 4, DDT	ND–42.76	1.58 ± 0.3

The detection levels were from not detected for Dimethotes (detection limit = 0.001 μg/kg), to 4041.73 μg/kg dry weight for Endosalfan I recorded in the present study (Table 1).

The concentrations of Endosalfan and its metabolites in fish muscle were recorded up to a mean of 341.50 ± 32.19 μg/kg. Bagumire et al. (2008) discovered a lower level (2 μg/kg) of Endosulfan in fish muscle in Uganda; Polder et al. (2014) discovered Endosulfan levels ranging from ND to 405 μg/kg in Tanzanian fish muscle. Deribe et al. (2014) discovered Endosalfan, ND–42.5 μg/kg in fish muscle from Lake Hawassa, Ethiopia, which is lower than the current result. In Gana, a study conducted by Kuranchie‐Mensah et al. (2013) reported an Endosulfan concentration of 0.01–7.52 μg/kg. Another study in Bénin by Yehouenou et al. (2014) noted Endosulfan of 9–215 μg/kg concentration.

The mean concentration of 4, 4, DDT and its metabolites in our study ranged from 1.58 to 64.01 μg/kg (Table 1). Similar to our finding, the study done by Deribe et al. (2014) in fish muscle of Lake Hawassa, Ethiopia detected a mean concentration range of 19–56 μg/kg of 4, 4, DDT. Another comparable study was done by Yohannes et al. (2014) on edible fish species from a Rift Valley Lake–Lake Ziway in Ethiopia, where the concentration of 4, 4, DDT was 0.77–61.9 μg/kg. Various studies conducted by different scholars worldwide, including Gustav Gbeddy et al. (2012) in Ghana, discovered that the mean DDT residue concentration in fish muscles ranges from 10 to 1700.35 μg/kg; another study conducted by Adu‐Kumi et al. (2010) in Ghanaian lakes reported up to 440.90 μg/kg; and Polder et al. (2014) in Tanzania recorded 7.2–319 μg/kg, indicating that the concentration levels of 4, 4, DDT were higher than our finding. A study done in Sri Lanka with an average of 735 μg/kg; 4, 4, DDT was detected, which was higher than the present finding (Bedigama & Gabadage, 2018). Lower than our findings were detected in a study done by Kasozi et al. (2006), that DDTs in fish samples from Lake Victoria, Uganda were 0.80–0.86 μg/kg. Another lower concentration of dry weight DDTs in fish muscle was reported in African studies: 0.70–0.90 μg/kg in Egypt by Yahia and Elsharkawy (2014), 3.88–11.3 μg/kg in Bénin by Yehouenou et al. (2014), and 2.098 μg/kg in Kenya by Omwenga et al. (2016).

This high occurrence in our study area might be due to the long‐term use of organochlorine pesticides to improve agricultural productivity in the catchments of the lake. The high lipophilicity, bioaccumulation nature, long half‐life, and potential for long‐range leaching nature of the chemicals result in the accumulation of persistent toxic substances in soil, water, and air (Agrawal et al., 2010; Ravindran et al., 2016; USEPA, 2009). The tropical conducive pests' multiplication environment of the water shed of Lake Tana might lead to the use of OCPs frequently and indiscriminately by the farmers (Agmas & Adugna, 2020). In addition, due to lack of proper implementation of legislation, improper market regulations, poor knowledge and skill of safe use of OCPs and ignorance shown by the farmers, agricultural workers in the Lake Tana catchment may be prone to experiencing high levels of agricultural chemicals, including OCPs (Agmas & Adugna, 2020; Beyene et al., 2016; Mengistie et al., 2016).

According to our findings, the detected level of 4, 4, DDT was lower than its metabolites (4, 4, DDE and 4, 4, DDD), and the Endosalfan sulfate concentration in fish muscle was lower than those of its metabolites (Endosalfan I and Endosalfan II) (Table 1). The lower concentration of the parent compounds may reflect the current limited use of those persistent OCPs in the Lake Tana catchments. The detected concentration may be due to a previous historic event with a long half‐life (2–15 years) (Jayaraj et al., 2016). However, a non‐negligible concentration of parent compounds of these persistent pollutants was detected, which indicates the continued use of these OCPs in the water shed of Lake Tana.

**FIGURE 2:**
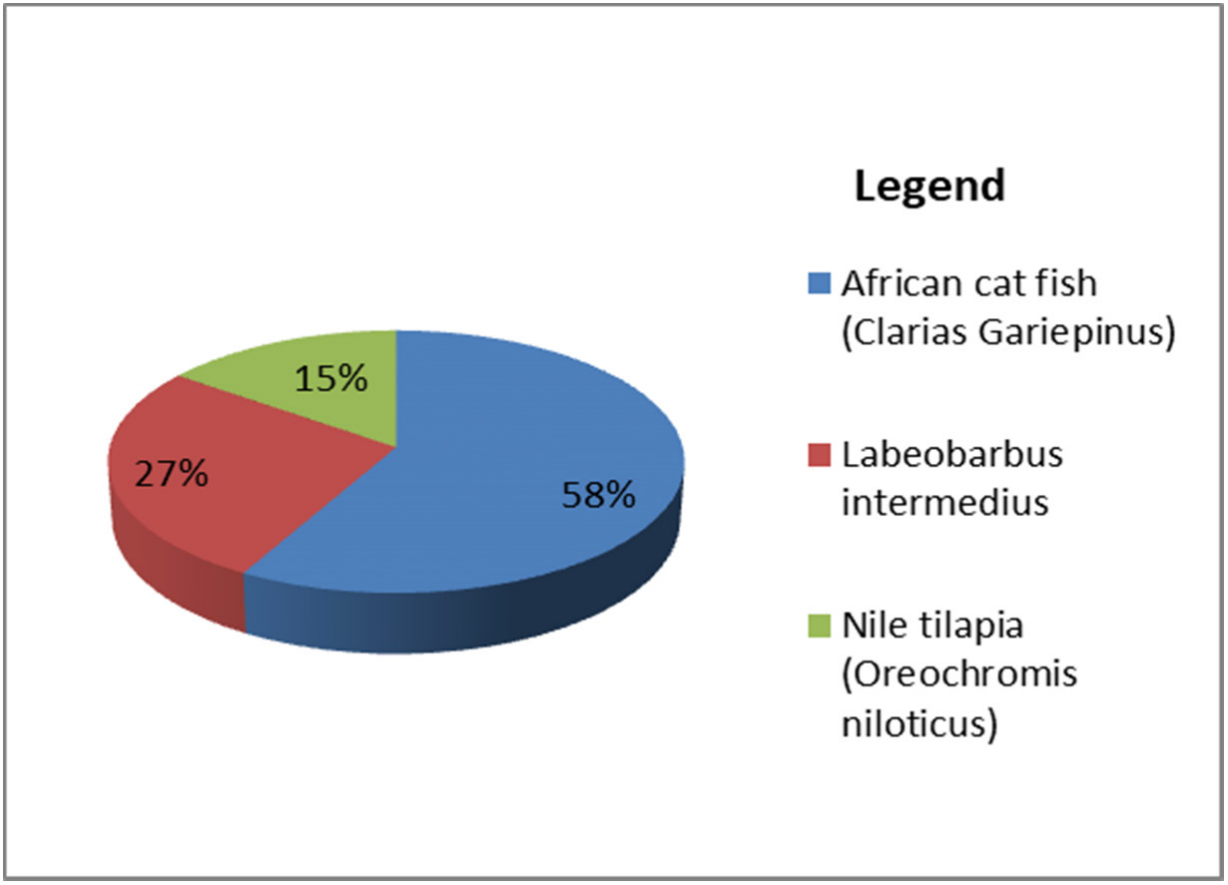
Comparison of organochlorine pesticides (OCPs) detected with fish groups

This study highlights that more pesticide was detected from African catfish (*Clarias gariepinus*) species than from other main commercially important fish groups (*Labeobarbus intermedius* and *O. niloticus*). Among all the covariances which were imported into multivariable logistic regression analysis, fish species and sampling sites of the study lake were independent variables significantly associated with the occurrence of pesticide (Figure 2).

The odds of having pesticide were 3.87 times higher in *Clarias gariepinus* species than in other commercially important fish species, *O. niloticus* (AOR = 3.87; 95% CI: 1.37–6.96). Similar to our finding, the study conducted by Nicklisch et al. (2017) observed high variation in pesticide levels when examining different fish species.

Spatially, occurrences were frequent at the Gorgora, Enferanze, and Bahir Dar sampling sites of the study lake. The odds of Gorgora sampling sites’ fish were 1.58 times more likely to have pesticide residue detected than those sampled fish from Delgie (AOR = 1.58; 95% CI: 1.33–1.99). This variable distribution of OCPs in our study area might be due to the environment as well as biological factors like amounts of lipid in their bodies and differences in ecological characteristics of the fish species (Borga et al., 2004). All the other measured risk factors were not statistically significant in our study. However, different studies have found that factors such as fish length, fish species, trophic level, habitat type, feeding habitat, and fish movement patterns and residence time in contaminated areas influence OCP pollutant levels in fish muscle (Bonito et al., 2016; Borga et al., 2004; Cardona, 2015; USEPA, 2009).

### 3.2 Public health risk

**FIGURE 3:**
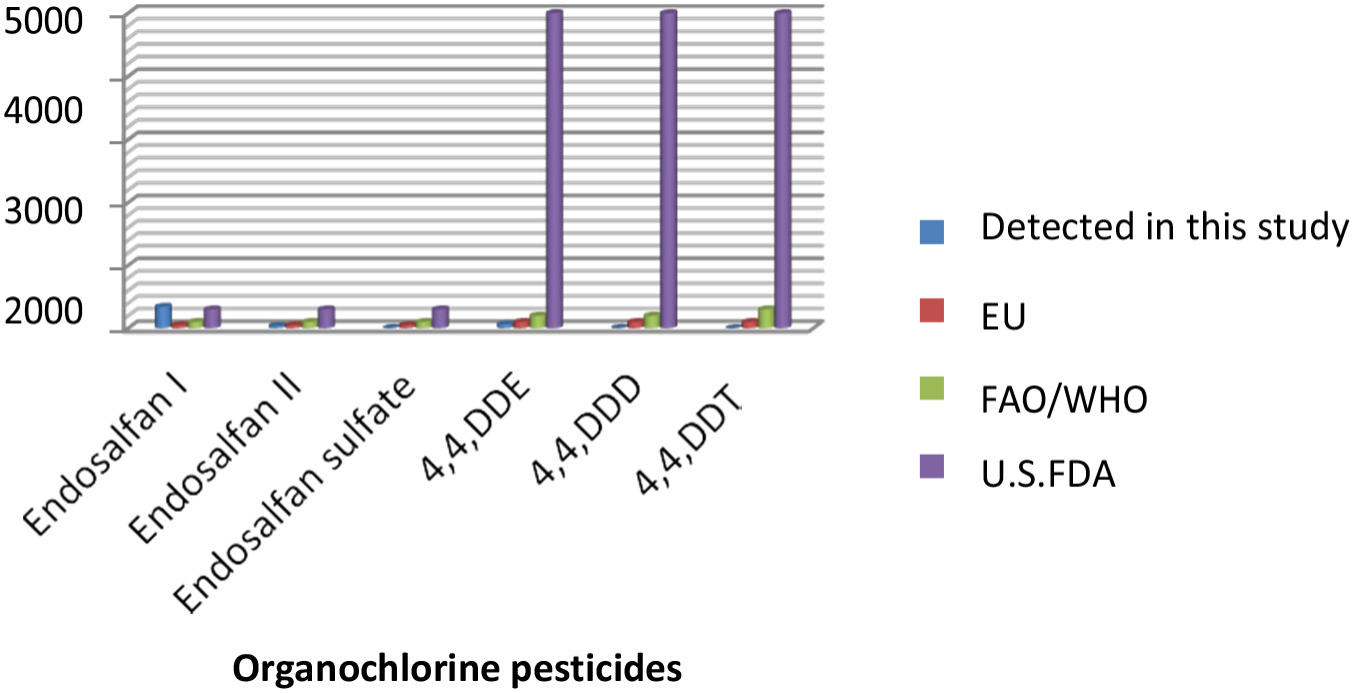
Comparison of organochlorine pesticides (OCPs) detected with maximum residue level (MRL) of different standards

Humans represent one of the top receptors for aquatic contaminants, and consumption of fish with high levels of contaminants may pose public health concerns. The consumption of contaminated fish may lead to serious public health problems if the concentration level is higher than the maximum residue level (MRL) and acceptable daily intake (ADI) standards of FAO/WHO (2009); U.S. Food and Administration (2009); and European Union (EU) (2013). The amount of OCPs residue concentration found in some fish meat samples in our study was higher than the recommended MRL of different standards. However, except for Endosalfan I, the overall mean value of concentration detected in our study was lower than the MRL standards of FAO/WHO (2009), U.S. Food and Administration (2009); and EU (2013) (Figure 3).

These higher concentrations of Endosalfan I in fish muscle originated from Lake Tana North West Ethiopia, indicating that it is alarming to the people who consume fish food.

Another method that health risk assessment of consumers from the intake of OCPs contaminated fish was done by calculating the health risk index (HRI). In our findings, the mean concentration detected was higher than the acceptable daily intake of WHO/FAO recommended value. The acceptable daily intake of Endosalfan I, Endosalfan II, 4, 4, DDE, and 4, 4, DDD recommended by WHO/FAO (2009) is 20, 20, 5, and 5 μg/kg, respectively. Due to the low per capita consumption rate of fish in Ethiopia, the present finding of a HRI ranges from 0.002 to 0.1275, indicating the present level has no public health risk (Table 2).

**TABLE 2:** ADIs, EDIs of organochlorine pesticides (OCPs) residues, and their HRIs associated with the consumption of fish from the Lake Tana

Pesticides	Range (μg/kg)	Mean ± SD	ADI	EDI	HRI	Health risk
Endosalfan I	0.001–4041.73	341.50 ± 32.19	20	2.55	0.1275	No
Endosalfan II	ND‐628.27	36.01 ± 2.3	20	0.27	0.0135	No
Endosalfan sulfate	ND‐146.69	5.43 ± 4.06	20	0.04	0.1275	No
4, 4, DDE	ND‐227.11	64.01 ± 9.08	5.0	0.48	0.096	No
4, 4, DDD	ND‐55.59	5.65 ± 3.12	5.0	0.04	0.008	No
4, 4, DDT	ND to 42.76	1.58 ± 0.301	5.0	0.01	0.002	No

Abbreviations ADI: acceptable daily intake, EDI: estimated daily intake; HRI health risk index; SD: Standard deviation; ND: not detected

But these values may pose a potential public health risk if the HRI is calculated by considering local per capita consumption. This minimum exposure with no public health risk (HRI < 1) was recorded in different studies, for instance in Ghana by Gbeddy et al. (2012) and Akoto et al. (2016). Similar to our finding, HRI of Endosalfan I, Endosalfan II, 4, 4, DDE, and 4, 4, DDD residues in fish of Edko Lake, Egypt did not have a public health risk (HRI < 1) (Abbassy et al., 2021). Another study by Hasan et al. (2022) in Bangladesh detects less than 1 HRI organochlorine pesticide in fish. In contrast to our finding, the study done by Yohannes et al. (2014) on a Rift Valley Lake‐Lake in Ziway, Ethiopia, indicated a potential cancer risk from consumption of the fish from the calculated hazard ratio (HR). Another study in Sri Lanka by Bedigama and Gabadage (2018) found HRI values > 1.

As a limitation of this study, it is necessary to conduct further studies that assess the detection of OCPs residues in human serum to examine the burden of these OCPs and integrating biomarkers of exposure and health risk effects, and modeling exposure routes via fish consumption.

## 4 CONCLUSION AND RECOMMENDATIONS

The present research finding showed that high concentrations of Endosalfan I residues in fish muscle were detected in fish samples, which are higher than the standard limit level. However, as a result of the low consumption rate of fish meat in the meantime, it will have little or no significant adverse public health effects on consumers. So, applying a more conservative threshold limit (WHO/FAO standard) implementation is a necessary action by considering health risks of local consumers, other sources of exposure, and vulnerable populations (children and pregnant women). The study highlights the presence of a non‐negligible level of OCPs in the muscle of fish that indicates there is still use of these pesticides in the catchments of Lake Tana, North West Ethiopia. As a result, one health intervention is recommended to protect against adverse effects on flora and fauna of the entire eco‐system as well as public health hazards. Collaborative work on awareness creation about the possible occurrence of OPCs residues should also be done among environmental health, agricultural, and public health offices. In addition, further study of the OCP residue in other agricultural food items and its effects on the ecology of the aquatic environment is recommended.

## FUNDING INFORMATION

This work was supported by the Ethiopian Institute of Agricultural Research, National Fishery and Aquatic Life Research Center [17.2/0409/2018], and Bahir Dar University [1/3040/1.2.9].

## CONFLICT OF INTEREST

The authors declare that they have no competing interests.

## ETHICAL APPROVAL

The study was granted an exemption from requiring ethics approval from the College of Agriculture and Environmental Science, Research Ethics and Review Board.

## CONSENT FOR PUBLICATION

Not applicable.

## Data Availability

The datasets used and/or analyzed this study are available from the corresponding author on reasonable request. The data are not publicly available due to privacy or ethical restrictions.
